# Prévalence et facteurs associés à l’hyperuricémie au cours du diabète de type 2 en milieu hospitalier Dakarois (Sénégal)

**DOI:** 10.11604/pamj.2025.51.45.47695

**Published:** 2025-06-16

**Authors:** Ngoné Diaba Diack, Nafy Ndiaye, Ali Hedroj, Arnaud Ndi Manga, Mouhamed Dieng, Djiby Sow, Michel Assane Ndour, Yakham Mouhamed Leye, Demba Diedhiou, Anna Sarr, Maimouna Ndour Mbaye, Abdoulaye Leye

**Affiliations:** 1 Service d'Endocrinologie-Diabétologie-Nutrition, Centre Hospitalier National de Pikine, Dakar, Sénégal,; 2 Faculté de Médecine, Université Cheikh Anta Diop, Dakar, Sénégal,; 3 Centre du Diabète Marc Sankalé, Hôpital Abass Ndao, Dakar, Sénégal,

**Keywords:** Hyperuricémie, diabète de type 2, magnésium, Sénégal, Hyperuricemia, type 2 diabetes, magnesium, Senegal

## Abstract

**Introduction:**

l'hyperuricémie augmente la morbi-mortalité cardiovasculaire au cours du diabète de type 2 (DT2). L'objectif de cette étude était de déterminer la prévalence de l'hyperuricémie asymptomatique dans une population de diabétique de type 2 ainsi que ses déterminants.

**Méthodes:**

il s'agissait d'une étude transversale menée au Centre Hospitalier National de Pikine et au Centre Hospitalier Abass Ndao (Dakar, Sénégal). Elle concernait des diabétiques de type 2 suivis en ambulatoire sans antécédents et/ou symptomatologie de goutte. Des prélèvements à jeun étaient effectués pour le dosage des paramètres métaboliques (acide urique, HbA1c, exploration lipidiques, magnésémie).

**Résultats:**

nous avions inclus 90 patients. Les patients étaient âgés en moyenne de 59,6 ± 8,8 ans. Il s'agissait de 24 hommes et 66 femmes. Le diabète évoluait depuis 5 ans en moyenne avec une médiane d'HbA1c à l'inclusion à 7,2%. La prévalence de l'hyperuricémie était de 31,1% (n=28). Après analyse multivariée, les facteurs indépendamment associés à l'hyperuricémie étaient le sexe masculin (odds ratio ajusté ORa: 3,9, IC à 95%: 1,3-12,1; p=0,015), un DFG inférieur à 60ml/min (ORa: 7,7, IC à 95%: 1,2-48,3; p=0,029). Une corrélation positive et significative était retrouvée entre le taux de triglycérides et l'uricémie (r=0,5; p< 0,001). La magnésémie était inversement corrélée à l'uricémie (r=-0,3; p=0,051).

**Conclusion:**

l'hyperuricémie est fréquente chez les diabétiques de type 2. Il est nécessaire de la dépister en particulier chez le sujet de sexe masculin, présentant une altération de la fonction rénale et/ou une hypertriglycéridémie.

## Abstract (en)

**Introduction:**

hyperuricemia increases cardiovascular morbidity and mortality in type 2 diabetes (T2D). The purpose of this study was to determine the prevalence of asymptomatic hyperuricemia in a population with type 2 diabetes and its determinants.

**Methods:**

we conducted a cross-sectional study at the Pikine National Hospital Center (CHNP) and the Abass Ndao Hospital Center (Dakar, Senegal). It involved outpatients with type 2 diabetes and without a history or symptoms of gout. Fasting blood samples were taken to measure metabolic parameters (uric acid, HbA1c, lipid profile, and magnesium levels).

**Results:**

the study included 90 patients. The mean age was 59,6 ± 8,8 years. There were 24 men and 66 women. The average duration of diabetes was 5 years, with a median HbA1c at inclusion of 7.2%. The prevalence of hyperuricemia was 31.1% (n=28). After multivariate analysis, the factors independently associated with hyperuricemia were male sex (adjusted odds ratio [aOR]: 3.9, 95% CI: 1,3-12,1; p=0,015), and GFR below 60 ml/min (aOR: 7.7, 95% CI: 1,2-48,3; p=0,029). A significant positive correlation was found between triglyceride levels and uric acid levels (r=0.5; p<0.001). Magnesium levels were inversely correlated with uric acid levels (r=-0,3; p=0,051).

**Conclusion:**

hyperuricemia is common in patients with type 2 diabetes. Screening is necessary, especially in male patients with impaired renal function and/or hypertriglyceridemia.

## Introduction

Le diabète de type 2 (DT2) constitue un véritable problème de santé publique en Afrique [1]. Sa prévalence a connu une hausse considérable ces dernières années [2,3]. Dans nos régions d'Afrique sub-saharienne, le DT2 est caractérisé par une prévalence élevée des complications vasculaires [1]. Le risque cardiovasculaire majeur associé au DT2 est en partie lié à son association fréquente à d'autres facteurs de risque cardiovasculaires, notamment l'hyperuricémie. En effet, au cours du DT2, l'hyperuricémie constitue un facteur de risque significatif de maladie coronarienne et d'une morbi-mortalité cardiovasculaire élevée [4]. Également, les patients diabétiques présentant une hyperuricémie sont plus à risque de développer des complications chroniques du diabète telles que la néphropathie diabétique qui accroît le risque de mortalité chez ces patients [5]. L'association DT2 et hyperuricémie est ainsi délétère pour les patients, du fait de la morbi-mortalité importante qu'elle entraîne.

La prévalence de l'hyperuricémie chez les diabétiques de type 2 est élevée [6]. Cette prévalence est toutefois variable en fonction des populations et des méthodes utilisées [7,8].

Le dépistage systématique de l'hyperuricémie au cours du DT2 est judicieux au vue de ces données. Toutefois, dans notre contexte, un dépistage systématique est difficile à promouvoir en raison des obstacles financiers et logistiques auxquelles nous sommes confrontés dans la gestion du diabète [1]. L'identification de facteurs de risque pourrait nous permettre dans ce contexte de réaliser un dépistage ciblé, adapté à nos populations.

C'est dans ce cadre que nous avions initié cette étude dont l'objectif principal était de déterminer la prévalence de l'hyperuricémie asymptomatique au cours du DT2 en milieu hospitalier Dakarois. Également, il s'agissait d'identifier les facteurs de risque qui lui y sont associés en vue d'un dépistage ciblé.

## Méthodes

**Plan et cadre d'étude:** nous avions mené une étude transversale descriptive à visée analytique. Elle s'était déroulée entre le 1^er^ avril et le 31 juin 2024. Cette étude avait pour cadre les deux plus grandes structures de prise en charge du diabète à Dakar (Sénégal): le service d'Endocrinologie-Diabétologie-Nutrition du Centre Hospitalier National de Pikine et le Centre du Diabète Marc Sankalé du Centre Hospitalier Abass Ndao. Tous les dosages biologiques étaient réalisés au Laboratoire de Biochimie du Centre Hospitalier National Dalal Jamm (Dakar, Sénégal).

**Population d'étude:** nous avions inclus toutes les personnes vivant avec un DT2 reçus en ambulatoire cette période et ayant librement consenti de participer à l'étude. Les patients présentant un antécédent de goutte, une goutte active ou une néphropathie uratique étaient exclus de l'étude. La formule de Cochrane a été utilisée pour le calcul de la taille d'échantillon.


$$N=Z^{2}P^{\left(1-P\right)}/e^{2}$$

La prévalence du diabète était estimée à 3,1% en se basant sur les données de *l'International Diabètes Federation* (IDF) de 2021 [2], avec un niveau de confiance à 95% et une marge d'erreur à 5%. C'est ainsi que nous avons obtenu une taille minimale de 46 participants pour la réalisation de cette étude. Un échantillonnage de convenance a été réalisé pour le recrutement des patients.

**Collecte de données:** les participants étaient abordés durant les consultations externes afin de leur présenter l'étude et obtenir leur consentement libre et éclairé. Les différentes données socio-démographiques et cliniques étaient recueillies. Ensuite, un rendez-vous avait été fixé à leur convenance pour le dosage des paramètres biochimiques. Des prélèvements de sang veineux ont ainsi été réalisés au pli du coude chez tous les patients inclus. Ces prélèvements étaient réalisés après un jeun d'au moins 9 heures de temps. Les échantillons étaient par la suite collectés et acheminés au Laboratoire de Biochimie du Centre Hospitalier National Dalal Jamm pour les différents dosages. Il s'agissait du dosage de l'uricémie, de l'hémoglobine glyquée (HbA1c), de la glycémie à jeun, de la créatininémie, de l'urémie, de la magnésémie et de l'exploration des anomalies lipidiques: dosage cholestérol total, triglycérides, *high density lipoprotein (HDL), low density lipoprotein (LDL)*.

**Variables:** les patients inclus étaient classés en 2 groupes: hyperuricémie+ (uricémie supérieure à 70mg/L chez les hommes et 60mg/L chez les femmes) et hyperuricémie- (uricémie inférieure à ces seuils). Chez tous les participants l'indice de masse corporelle (IMC) était calculé selon l'indice de Quételet et stratifié selon le système de classification de l'obésité *Edmonton Obesity Staging System* (EOSS). Ainsi le surpoids était défini par un IMC supérieur ou égal à 25kg/m^2^ et la maigreur par un IMC inférieur ou égal à 18,5kg/m^2^. L'obésité abdominale était définie selon les critères harmonisés de 2009 du syndrome métabolique [9]. Nous avions considéré que le DT2 était déséquilibré lorsque l'HbA1c était supérieure ou égale à 7,5%. Le débit de filtration glomérulaire (DFG) était calculé selon la formule Chronic Kidney Disease Epidemiology Collaboration" (CKD-Epi) et exprimé en ml/min/1,73 m^2^. Une hypomagnésimie était définie par un taux de magnésium sanguin inférieur à 0,65mmol/L.

**Analyse statistique:** les données ainsi recueillies ont été saisies et analysées grâce au logiciel Statistical Package for the Social Sciences (SPSS) version 27.0. La régression logistique simple a été utilisée pour la recherche des facteurs associés à l'hyperuricémie. L'odds ratio (OR) a été utilisé comme mesure d'association assorti de son intervalle de confiance (IC) à 95%. Une analyse multivariée a ensuite été réalisée. Les variables qui se sont avérées significatives dans la régression logistique simple ont été introduites l'une après l'autre progressivement dans le modèle multivarié pour voir celles qui étaient indépendamment associées à l'hyperuricémie. Cette analyse a permis de déterminer l'odds ratio ajusté (ORa). Également, le coefficient de Pearson était utilisé pour établir les corrélations entre le taux de triglycérides, de magnésium et l'uricémie. Le test T de Student et le test de Mann-Whitney étaient utilisés respectivement pour comparer les moyennes et les médianes. Nous avions considéré une valeur de P-value (p) < 0,05 comme significative.

**Considérations éthiques:** cette étude ne comportait aucun risque pour les participants. L'anonymat et la confidentialité étaient de mise durant la réalisation de cette étude. Seuls les membres de l'équipe de recherche avaient accès aux informations sur les participants à cette étude. Tous les participants avaient signé une fiche de consentement libre et éclairé. Par ailleurs, le protocole de l'étude était au préalable validé par le comité local d'éthique du Centre Hospitalier National de Pikine.

## Résultats

**Caractéristiques générales de la population d'étude:** durant la période de collecte des données, 113 participants ont été approchés dans les 2 services. Parmi eux, 18 ont refusé de participer à l'étude et 5 ont été exclus pour goutte. Au total, nous avions inclus 90 patients. L'âge moyen de la population d'étude était de 59,6 ± 8,8 ans avec des extrêmes de 37 et 77 ans (Tableau 1). Cette population était constituée de 24 hommes et 66 femmes. Ils étaient diabétiques de type 2, depuis en moyenne 5 ans. Ils recevaient un traitement antidiabétique non insulinique dans 61% des cas. L'insulinothérapie était utilisée chez 32,2% (n=29) de la population. L'HbA1c à l'inclusion était en moyenne de 7,1%. A l'inclusion, le diabète était déséquilibré dans 40% des cas (n=36). Dans cette population, l'IMC était en moyenne de 22,8 ± 3,2kg/m^2^. D'autres facteurs de risque cardiovasculaires étaient associés au DT2. Il s'agissait essentiellement de l'hypertension artérielle (HTA) retrouvée chez 47% des participants (n=43), de la dyslipidémie présente chez 61,1% (n=55). Le surpoids et l'obésité abdominale étaient notés respectivement dans 30 (n=27) et 57,8% (n=52) des cas. Sur le plan biologique, un taux de créatinine anormal (supérieur à 13mg/L) et d'urée (supérieur à 0,45g/l) étaient retrouvés respectivement chez 10 et 13 participants. Une altération de la fonction rénale avec un DFG inférieur à 60ml/min était retrouvée dans 13,3% des cas (n=12). En outre, nos patients présentaient une hypomagnésémie dans 65,6% des cas (n=59).

**Tableau 1:** moyennes d'âge, d'indice de masse corporelle (IMC), de tour de taille et de pression artérielle (PA) de notre population d'étude

Variables Moyenne ± Écart type	Total	Hyperuricémie (+)	Hyperuricémie (-)	P-value
**Age** (année)	59,6 ± 8,8	59 ± 9,9	59,9 ± 8,2	0,662
**IMC** (Kg/m^2^)	22,8 ± 3,2	23,5 ± 2,7	22,5 ± 3,4	0,176
**Tour de taille** (cm)	87,8 ± 11,7	90 ± 10,4	86,8 ± 12,2	0,241
**PAS** (mmHg)	134,3 ± 11,1	136,3 ± 13	133,4 ± 10,1	0,251
**PAD** (mmHg)	82,1 ± 10,3	84 ± 9,6	81,3 ± 10,5	0,260

IMC: indice de masse corporelle; PAS: pression artérielle systolique; PAD: pression artérielle diastolique

**Prévalence de l'hyperuricémie:** la prévalence de l'hyperuricémie asymptomatique au sein de cette population de diabétique de type 2 était de 31,1% (n=28). La médiane d'acide urique était de 76,5mg/L (65,7-84,2) dans le groupe Hyperuricémie+ contre 42,9mg/L (36,7-55,2) dans le groupe hyperuricémie (Tableau 2).

**Tableau 2:** médiane des paramètres biologiques dans notre population d'étude

Variables Médiane (IQ)	Total	Hyperuricémie (+)	Hyperuricémie (-)	P-value
Glycémie (g/L)	1,3 (1,1-1,6)	1,4 (1,1-1,7)	1,4 (1,1-1,6)	0,903
HbA1c (%)	7,2 (6,7-8)	7,3 (6,8-8)	7,1 (6,7-8)	0,618
**Acide urique** (mg/L)	54,7 (40,2-65,2)	76,5 (65,7-84,2)	42,9 (36,7-55,2)	< **0,001**
Magnésium (mg/L)	17,1 (15,4-18,6)	17,7 (15,6-19,6)	16,9 (15,2-18,5)	0,224
**Urée** (g/L)	0,3 (0,2-0,4)	0,4 (0,2-0,6)	0,2 (0,1-0,3)	< **0,001**
**Créatinine** (mg/L)	7,4 (6,2-9,7)	10,3 (7,9-14,5)	6,7 (6-7,9)	< **0,001**
**DFG** (ml/min/1,72 m2)	101,1 (75,2-113,1)	71,5 (53,6-99,8)	106,7 (93,6-116,2)	< **0,001**
**C-Total** (g/L)	1,9 (1,7-2,4)	2,2 (1,8-2,8)	1,9 (1,6-2,3)	**0,033**
C-HDL (g/L)	0,5 (0,4-0,6)	0,5 (0,3-0,6)	0,5 (0,4-0,6)	0,524
C-LDL (g/L)	1,2 (0,9-1,5)	1,4 (1-2)	1,2 (0,9-1,4)	0,142
**Triglycérides** (g/L)	0,8 (0,6-1,4)	1,2 (0,9-1,7)	0,7 (0,5-1,1)	< **0,001**

DFG: débit de filtration glomérulaire; C-Total: cholestérol total; C-HDL: cholestérol HDL; C-LDL: cholestérol LDL

**Figure 1:**
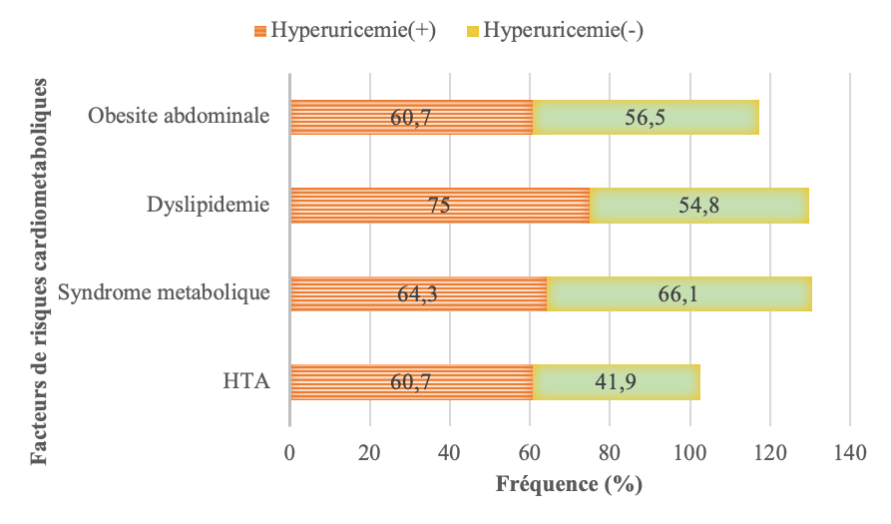
répartition des facteurs de risque cardio-métaboliques selon le statut d'uricémie

**Facteurs associés à l'hyperuricémie:** l'analyse bivariée entre l'hyperuricémie et les différents paramètres socio-démographiques, cliniques et biologiques est présentée au niveau du (Tableau 3). Sur le plan socio-démographique, il n'y avait pas de différence significative entre les moyennes d'âges dans les groupes hyperuricémie+ et hyperuricémie-: 59 ± 9,9 ans versus 59,9 ± 8,2 ans; p =0,987. Le sexe masculin était significativement associé à l'hyperuricémie dans notre étude (OR= 4, IC à 95%: 1,4-10,7; p= 0,006). Les patients présentant une hyperuricémie avaient une médiane d'HbA1c plus élevée que ceux sans hyperuricémie: 7,3% (6,8-8) versus 7,1% (6,7-8); p = 0,618 (Tableau 2). Le caractère déséquilibré du diabète n'avait pas d'influence significative sur la présence d'une hyperuricémie (OR=1,2, IC à 95%: 0,4-2,9; p= 0,70). L'insuline était utilisée chez 20 patients présentant une hyperuricémie contre 9 patients sans hyperuricémie (p= 0,991). Tous les autres facteurs de risque cardiovasculaires associés au DT2 dans notre étude étaient plus fréquents chez les patients présentant une hyperuricémie. La (Figure 1) présente la répartition des facteurs de risque cardiovasculaires retrouvés dans notre population d'étude en fonction du statut d'hyperuricémie. Le surpoids concernait plus les patients diabétiques de type 2 présentant une hyperuricémie (32,1% versus 29% ; p=0,766). Le phénomène inverse était observé en ce qui concerne la maigreur (3,6% versus 12,9%). Les moyennes du tour de taille étaient respectivement dans les groupes avec ou sans hyperuricémie de 90 ± 10,4 versus 86,8 ± 12,2; p= 0,241 (Tableau 1). L'hypercholestérolémie concernait 57% des patients avec hyperuricémie et 41,9% des patients sans hyperuricémie. L'augmentation du LDL cholestérol retrouvée chez la moitié des patients présentant une hyperuricémie était notée dans 38,7% des cas dans la population sans hyperuricémie. La moyenne de triglycérides était respectivement de 1,2g/L et 0,7g/L dans les groupes hyperuricémie+ et hyperuricémie. L'association entre la présence des autres facteurs de risque cardiovasculaire tels que l'HTA, la dyslipidémie, le surpoids, l'obésité abdominale et l'existence d'une hyperuricémie n'était pas significative (Tableau 3).

**Tableau 3:** facteurs associés à l’hyperuricémie dans une population de diabétiques de type 2 (n=90) entre janvier et juillet 2024 en milieu hospitalier Dakarois (Sénégal)

Variables	Risque d’hyperuricémie			
	Rapport des cotes non ajusté-RC (IC -95%)	P-value	Rapport des cotes ajusté-Rca (IC -95%)	P-value
**Sexe**	-	-	-	-
**Masculin**	4 (1,4 -10,7)	0,006	3,9 (1,3 -12,1)	0,015
**Féminin**	-	-	-	-
**Tranche d´âge**	-	-	-	-
[35 -50[	1,4 (0,3 -5,7)	0,587	-	-
[50 -65[	0,9 (0,4 -2,6)	0,984	-	-
[65 -80]	-	-	-	-
**Facteurs de risque cardiovasculaires**	-	-	-	-
**HTA**	2,1 (0,8 -5,3)	0,099	-	-
**Surpoids**	1,1 (0,4 -3)	0,766	-	-
**Obésité-abdominale**	1,2 (0,4 -2,9)	0,705	-	-
**Dyslipidémie**	2,4 (0,9 -6,6)	0,069	-	-
**Syndrome métabolique**	1 (0,3 -2,3)	0,865	-	-
**Traitement antihypertenseur**	-	-	-	-
**IEC**	1,5 (0,6 -3,9)	0,331	-	-
**Thiazidique**	1,7 (0,3 -8,3)	0,673	-	-
**Traitement Antidiabétique**	-	-	-	-
**Insulinique**	1 (0,3 -2,5)	0,991	-	-
**Non insulinique**	-	-	-	-
**Anomalies biologiques**	-	-	-	-
**Glycémie à-jeun ≥ 1,80 g/L**	0,6 (0,2 -1,6)	0,403	-	-
**HbA1c ≥ 7,5%**	1,2 (0,4 -2,9)	0,710	-	-
**Hypomagnésémie**	0,6 (0,2 -1,4)	0,259	-	-
**Créatinine > 13 (mg/L)**	6,5 (1,5 -27,7)	0,009	-	-
**Urée > 0,45 (g/L)**	10,9 (2,7 -44)	< 0,001	4,5 (0,8 -24,1)	0,077
**DFG < 60 ml/min**	16,6 (3,3 -83,1)	< 0,001	7,7 (1,2 -48,3)	**0,029**

HTA: hypertension artérielle; IEC: inhibiteurs de l’enzyme de conversion; HbA1c: hémoglobine glyquée; DFG: débit de filtration glomérulaire

**Figure 2:**
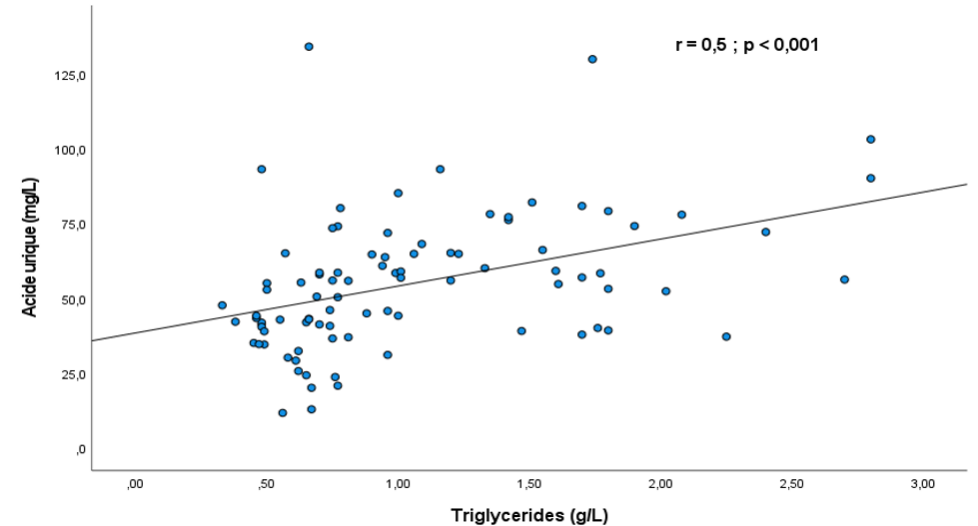
nuage de points représentant la variation du taux d'acide urique en fonction du taux de triglycérides

**Figure 3:**
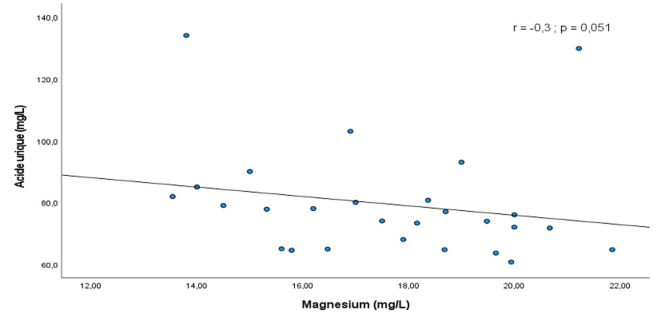
corrélation entre l'uricémie et la magnésémie chez les participants présentant une hyperuricémie

Concernant les paramètres rénaux, une altération de la fonction rénale avec un DFG inférieur à 60 ml/min était retrouvée chez 10 patients présentant une hyperuricémie sur les 12 de la population d'étude présentant cette anomalie. Globalement, le DFG était plus bas dans le groupe avec hyperuricémie comparativement au groupe sans hyperuricémie, avec une médiane respectivement à 71,5 (53,6-99,8) ml/min versus 106,7 (93,6-116,2) ml/min, p < 0,001. L'association altération de la fonction rénale avec un DFG inférieur à 60ml/min/1.73 m^2^ et hyperuricémie était statistiquement significative (OR= 16,6 ; IC à 95%: 3,3-83,1; p < 0,001). En outre, parmi les participants présentant une hypomagnésémie (n=59), 16 avaient une hyperuricémie et 43 n'en avaient pas, sans différence significative entre les deux groupes (p=0,259). Après analyse multivariée, les facteurs de risque indépendamment associés à l'hyperuricémie dans notre population d'étude étaient le sexe masculin (ORa: 3,9, IC à 95%: 1,3-12,1; p=0,015) et un DFG inférieur à 60 ml/min (ORa: 7,7, IC à 95%: 1,2-48,3; p=0,029). Par ailleurs, les tests de corrélations mettaient en évidence plusieurs associations significatives entre les paramètres biologiques. Une corrélation positive, significative et modérée (r=0,5; p <0,001) entre les taux d'acide urique et de triglycérides était ainsi notée (Figure 2). Cette corrélation était négative et significative (r=-0,3; p=0,051) entre l'uricémie et la magnésémie (Figure 3).

## Discussion

Nous avons mené une étude transversale dans une population de diabétique de type 2, afin de déterminer la prévalence de l'hyperuricémie asymptomatique ainsi que ses déterminants. La prévalence hospitalière de l'hyperuricémie était de 31% proche de celle retrouvée dans des travaux similaires menés sur le continent africain [7,10]. L'hyperuricémie chez les patients vivants avec le DT2 serait la conséquence de l'insulinorésistance qui d'une part entraine une diminution de l'excrétion urinaire d'acide urique et d'autre part une augmentation de la production de cette dernière via la voie de la purinosynthèse de novo [11]. Cette hyperuricémie contribue aux complications cardiovasculaires et rénales au cours du DT2. En effet, chez les patients diabétiques de type 2, l'hyperuricémie même asymptomatique est associée à une morbi-mortalité cardiovasculaire élevée [4]. Cette augmentation du risque cardiovasculaire au cours de l'hyperuricémie est aussi le fait de son association fréquente à d'autres facteurs de risque cardiovasculaire sur le terrain de DT2 [6,12]. Dans notre étude, la fréquence des facteurs de risque cardio-métaboliques était élevée au sein des patients présentant une hyperuricémie. Il s'agissait notamment de l'hypertension artérielle, de l'obésité mais surtout la dyslipidémie retrouvée chez 75% des patients. Nous avions d'ailleurs mis en évidence une corrélation positive et significative entre le taux d'acide urique et le taux de triglycérides. Dans la littérature, une hypertriglycéridémie a été identifiée comme facteur de risque du développer une hyperuricémie [13]. La pathogénie n'est pas complétement élucidée. Des théories récentes sous-tendent que l'accumulation d'acide urique au niveau des cellules pourrait entraîner une dysfonction mitochondriale à l'origine d'une libération de citrate dans le cytosol, initiant la lipogenèse et la synthèse des triglycérides [14]. D'autres facteurs de risque de survenue d'hyperuricémie au cours du DT2 sont identifiés dans ce travail. Après analyse multivariée, il s'agissait principalement du sexe masculin et la présence d'une altération de la fonction rénale avec un DFG < 60ml/min. Par contre il n'y avait pas de relation significative avec les niveaux d'hyperglycémie et l'hyperuricémie. Les participants de notre étude ayant une HbA1c supérieure ou égale à 7,5% avaient certes un risque plus élevé de développer une hyperuricémie mais cette association n'était pas significative. En outre, notre étude a mis en évidence une corrélation négative mais non significative entre la magnésémie et l'uricémie. Il faut rappeler que le magnésium est un oligoélément qui a montré des effets bénéfiques sur la réduction du taux d'acide urique via l'altération des mécanismes inflammatoires [15].

Nous pouvons retenir que l'association DT2 et hyperuricémie est fréquente dans notre contexte. L'hyperuricémie du fait de son association à une morbi-mortalité cardiovasculaire élevée doit être recherchée chez les diabétiques de type 2. En particulier, ceux de sexe masculin ou présentant une altération de la fonction rénale. Le taux de triglycérides pourrait également être un bon paramètre pour la prédiction d'une hyperuricémie chez les personnes vivants avec le DT2 en raison de la corrélation significative et positive entre ces 2 paramètres. Cependant, bien que l'hypomagnésémie soit fréquente chez les patients diabétiques de type 2 présentant une hyperuricémie, son implication clinique réelle reste à être déterminée.

Nous avons ainsi réalisé une étude prospective dans une population de 90 diabétiques de type 2. Cette étude est l'une des premières dans notre contexte à déterminer la prévalence de l'hyperuricémie ainsi que les facteurs qui lui sont associés. Elle a mis en évidence une prévalence élevée de l'hyperuricémie en milieu hospitalier Dakarois, en accord avec des travaux similaires en Afrique ainsi que dans le monde [7,10]. Toutefois, le caractère transversal de l'étude ne permet pas d'établir la chronologie d'installation de l'hyperuricémie par rapport aux autres anomalies biologiques. Ceci a notamment limité l'étude des relations entre l'hyperuricémie et les niveaux glycémiques chez ces patients. Ainsi, dans les perspectives de ce travail, il serait donc utile de suivre ces patients sur le long terme afin d'étudier les relations entre l'hyperuricémie et les autres paramètres métaboliques et donc de mieux cibler le dépistage. Au-delà du dépistage, la question du bénéfice du contrôle de l'hyperuricémie asymptomatique sur le risque cardiovasculaire se pose dans nos populations africaines. Il s'agit d'un chantier d'investigations futures.

## Conclusion

L'hyperuricémie est une condition fréquente chez les diabétiques de type 2. Notre étude a confirmé la prévalence élevée de cette association. Dans notre contexte, il est nécessaire de dépister l'hyperuricémie chez le diabétique de type 2 en particulier chez le sujet de sexe masculin, présentant une altération de la fonction rénale et/ou une hypertriglycéridémie. Cette étude ouvre également des perspectives sur les liens entre hyperuricémie et hypomagnésémie. Ainsi, la supplémentation en magnésium est une piste prometteuse qu'il est utile d'explorer chez nos populations dans le futur.

### Etat des connaissances sur le sujet

- L'association hyperuricémie et diabète de type 2 est fréquente;
- L'hyperuricémie est associé au déséquilibre du diabète;
- Rareté des études sur ce sujet au Sénégal.

### Contribution de notre étude à la connaissance

- La prévalence de l'hyperuricémie au cours du diabète de type 2 en milieu hospitalier Dakarois est de 31%;
- Il n'y avait pas de corrélation significative entre l'hyperuricémie et le déséquilibre du diabète.

## References

[1] Motala AA, Mbanya JC, Ramaiya K, Pirie FJ, Ekoru K (2022). Type 2 diabetes mellitus in sub-Saharan Africa: challenges and opportunities. Nat Rev Endocrinol.

[2] Sun H, Saeedi P, Karuranga S, Pinkepank M, Ogurtsova K, Duncan BB (2022). IDF Diabetes Atlas: Global, regional and country-level diabetes prevalence estimates for 2021 and projections for 2045. Diabetes Res Clin Pract.

[3] NCD Risk Factor Collaboration (NCD-RisC) (2024). Worldwide trends in diabetes prevalence and treatment from 1990 to 2022: a pooled analysis of 1108 population-representative studies with 141 million participants. Lancet.

[4] Venishetty S, Bhat R, Rajagopal KV (2018). Serum Uric Acid Levels in Type 2 Diabetes Mellitus: Is There a Linear Relationship with Severity of Carotid Atherosclerosis?. Indian J Endocrinol Metab.

[5] Shah P, Bjornstad P, Johnson RJ (2016). Hyperuricemia as a potential risk factor for type 2 diabetes and diabetic nephropathy. J Bras Nefrol.

[6] Ogbera AO, Azenabor AO (2010). Hyperuricemia and the metabolic syndrome in type 2 diabetes mellitus. Diabetol Metab Syndr.

[7] Alemayehu E, Fiseha T, Bambo GM, Sahile Kebede S, Bisetegn H, Tilahun M (2023). Prevalence of hyperuricemia among type 2 diabetes mellitus patients in Africa: a systematic review and meta-analysis. BMC Endocr Disord.

[8] Sun S, Chen L, Chen D, Li Y, Liu G, Ma L (2023). Prevalence and associated factors of hyperuricemia among Chinese patients with diabetes: a cross-sectional study. Ther Adv Endocrinol Metab.

[9] Alberti KGMM, Eckel RH, Grundy SM, Zimmet PZ, Cleeman JI, Donato KA (2009). Harmonizing the metabolic syndrome: a joint interim statement of the International Diabetes Federation Task Force on Epidemiology and Prevention; National Heart, Lung, and Blood Institute; American Heart Association; World Heart Federation; International Atherosclerosis Society; and International Association for the Study of Obesity. Circulation.

[10] Choukem SP, Mengue JA, Doualla MS, Donfack OT, Beyiha G, Luma HN (2016). Hyperuricaemia in patients with type 2 diabetes in a tertiary healthcare centre in sub-Saharan Africa: prevalence and determinants. Trop Doct.

[11] Crawley WT, Jungels CG, Stenmark KR, Fini MA (2022). U-shaped association of uric acid to overall-cause mortality and its impact on clinical management of hyperuricemia. Redox Biol.

[12] Woldeamlak B, Yirdaw K, Biadgo B (2019). Hyperuricemia and its Association with Cardiovascular Disease Risk Factors in Type Two Diabetes Mellitus Patients at the University of Gondar Hospital, Northwest Ethiopia. EJIFCC.

[13] Tan MY, Mo CY, Li F, Zhao Q (2023). The association between serum uric acid and hypertriglyceridemia: evidence from the national health and nutrition examination survey (2007-2018). Front Endocrinol (Lausanne).

[14] Ipsen DH, Lykkesfeldt J, Tveden-Nyborg P (2018). Molecular mechanisms of hepatic lipid accumulation in non-alcoholic fatty liver disease. Cell Mol Life Sci CMLS.

[15] Dibaba DT, Xun P, He K (2014). Dietary magnesium intake is inversely associated with serum C-reactive protein levels: meta-analysis and systematic review. Eur J Clin Nutr.

